# Effects of psychological support intervention on patients with nasopharyngeal carcinoma undergoing radiotherapy

**DOI:** 10.12669/pjms.40.7.7875

**Published:** 2024-08

**Authors:** Ce Wang, Xiaohui Liu, Yanhong Li, Dongxue Liu, Litao Yang, Yue Wang

**Affiliations:** 1 Ce Wang, Department of Radiotherapy, Affiliated Hospital of Hebei University, Baoding, Hebei, 071000, China; 2 Xiaohui Liu, Department of Radiotherapy, Affiliated Hospital of Hebei University, Baoding, Hebei, 071000, China; 3 Yanhong Li, Department of Radiotherapy, Affiliated Hospital of Hebei University, Baoding, Hebei, 071000, China; 4 Dongxue Liu, Department of Radiotherapy, Affiliated Hospital of Hebei University, Baoding, Hebei, 071000, China; 5 Litao Yang, Department of Orthopedics, Baoding NO.1 Central Hospital, Baoding, Hebei, 071000, China; 6 Yue Wang, Department of Radiotherapy, Affiliated Hospital of Hebei University, Baoding, Hebei, 071000, China

**Keywords:** Psychological support intervention, Nasopharyngeal tumor, Skin reaction, RTOG grading, QLQ-C30 score, Quality of life

## Abstract

**Objective:**

To find out the effects of psychological support intervention on patients with nasopharyngeal carcinoma undergoing radiotherapy.

**Methods:**

This was a retrospective study. Sixty six patients with nasopharyngeal carcinoma who received radiotherapy in the Affiliated Hospital of Hebei University from March 2021 to March 2022 were included and randomly divided into the observation group and the control group, with 33 cases in each group. Patients in the control group were given conventional care measures, while those in the observation group were given psychological support intervention on top of conventional care measures. The nursing effects between the two groups were compared.

**Results:**

After the intervention, the psychological resilience score of the observation group was significantly higher than that of the control group, with a statistically significant difference (P<0.05). The psychological resilience scores after the intervention were significantly higher in the observation group than before the intervention, and those in the control group were higher than before the intervention, with a statistically significant difference(P<0.05). The overall health score of quality of life in the observation group was significantly higher than that in the control group after the intervention, with a statistically significant difference(P<0.05). Moreover, the skin reaction in the observation group after radiotherapy was significantly better than that of the control group (P<0.01).

**Conclusion:**

Psychological support intervention is an effective means to treat patients with nasopharyngeal carcinoma, which results in various benefits such as improving patients’ mental resilience and quality of life and reducing the incidence of adverse reactions after radiotherapy.

## INTRODUCTION

Nasopharyngeal carcinoma (NPC), a highly prevalent malignancy in China, is one of the most common malignant tumors in southern China and Southeast Asia, with the highest incidence and morbidity and mortality rates among head and neck malignancies,^1^-^3^ WAN^4^ pointed out in a study that 19%-29% of NPC patients still had a local recurrence or distant metastases after radiotherapy, and once recurrence occurred, the five years survival rate was significantly reduced with an extremely poor prognosis. Given the special anatomical location, complex pathogenesis, and high risk of complications from surgical treatment, nasopharyngeal carcinoma is mainly treated with radiation therapy in clinical practice.^5^-^7^ Patients are vulnerable to physiological discomfort and psychological stress during radiation therapy due to the clinical symptoms of the disease and the treatment method, resulting in a decrease in quality of life and unsatisfactory treatment effects.^8^ Psychological resilience, which reflects the psychological changes of patients, mainly refers to the state of patients’ reactions to various types of problems. The higher the level of psychological resilience of patients, the more they can actively and effectively cope with various negative events in their lives. Therefore, psychological support intervention is of particular importance for the treatment of NPC patients, which can promote patients’ psychological health, reduce the adverse effects of radiotherapy, and improve psychological resilience and quality of life.^9^,^10^ In this study, the effects of psychological support intervention on patients with nasopharyngeal carcinoma undergoing radiotherapy were investigated.

## METHODS

This was a retrospective study. Sixty-six patients with nasopharyngeal carcinoma who received radiotherapy in the Affiliated Hospital of Hebei University from March 2021 to March 2022 were included and randomly divided into two groups according to the random number table method: the observation group and the control group, with 33 cases in each group.

### Ethical Approval:

The study was approved by the Institutional Ethics Committee of Affiliated Hospital of Hebei University (No.: HDFYLL-KY-2022-006; date: October 15, 2022), and written informed consent was obtained from all participants, patient privacy was guaranteed throughout the study.

### Inclusion criteria:

- Patients with nasopharyngeal carcinoma undergoing radiotherapy;
- Patients with KPS score >60;
- Patients who had basic thinking and judgment ability and could cooperate to complete the scale assessment.

### Exclusion criteria:

- Patients who abandoned treatment midway;
- Patients who were illiterate or cognitively impaired;
- Malignant tumors in other sites of the body at the same time.
- Patients in the control group were given conventional nursing intervention, and given routine medication and diet guidance, health education, and psychological care during postoperative radiotherapy.
- Patients in the observation group were given conventional nursing intervention combined with psychological support intervention methods.

### Psychological intervention:

One-on-one interview was adopted to make clear the psychological state of patients with nasopharyngeal carcinoma, and regular lectures were conducted on the knowledge of radiotherapy and related diseases to enhance the confidence of patients.

Short videos of radiotherapy-related knowledge were played, which were easy to understand and supported playback at any time.

### Narrative nursing approach:

Communication with patients was conducted at three points before, during, and after radiotherapy to establish a mutually trusting relationship. Patients were helped to find the problems that troubled them at each stage and were positively oriented, and positive feedback was used to try to solve problems, alleviate negative emotions and reduce anxiety. Simultaneously, patients were given companionship and encouragement to promote their confidence to continue treatment and strengthen their belief to fight against the disease and eventually overcome it and return to their families and society. Nursing staff can establish a good doctor-patient relationship with patients, win their trust and cooperation. During the six-months follow-up of this study, the survival rate was 100%.

### Observation indexes:

10-item Connor-Davidson Resilience Scale (CD-RISC);^11^ the scale is scored on a six point Likert scale, in which 0 indicates not at all, 1 indicates rarely, two indicates sometimes, 4 indicates often, and 5 indicates almost always, with a total score of 0-50. The higher the score, the higher the psychological resilience of the patient. The Cronbach’s alpha coefficient of the scale was 0.882, and the retest reliability was 0.743.

The EORTC Core Quality of Life questionnaire (EORTC QLQ-C30):^12^ the scale has 30 items set in the domains of functional domains, symptom domains, and general health status. All items are rated from 0-100, with a high score indicating good functioning and a high quality of life.

RTOG grading: the RTOG Radiation Damage Assessment Scale^13^ was utilized to grade and evaluate the adverse reactions after radiotherapy for nasopharyngeal carcinomas, such as skin reaction and nausea. Each item was graded on a scale of 1-4, with a score of 0-3, respectively, and the total score was the skin reaction score under radiotherapy. All the questionnaire was simply handed over to the participants to fill themselves.

### Statistical analysis:

All data in this study were statistically analyzed by SPSS 20.0 software. The count data were expressed as frequencies and percentages, and measurement data were expressed as (*χ̅*±*S*). Two independent sample t test was used for comparison between groups, and c^2^ test was used for the comparison of rates. P<0.05 indicates a statistically significant difference.

## RESULTS

According to the results of psychological support intervention on the psychological status of NPC patients during radiotherapy before and after the intervention, there was no statistically significant difference in psychological resilience scores between the observation group and the control group before the intervention (P>0.05), indicating comparability between the two groups. After the intervention, the psychological resilience score of the observation group was significantly higher than that of the control group, with a statistically significant difference (P<0.05). The psychological resilience scores after the intervention were significantly higher in the observation group than before the intervention, and those in the control group were higher than before the intervention, with a statistically significant difference (P<0.05) (Table-I).

**Table-I:** Psychological resilience scores before and after the intervention (*χ̅*±*S*).

Group	n	Before intervention	After intervention	t value	P value
Observation group	33	58.39±3.579	64.82±2.325	5.968	<0.001
Control group	33	52.12±3.480	54.00±3.509	6.210	<0.001
t value		2.24	4.303		
P value		0.54	<0.001		

*P<0.05.

According to the results of the two groups before and after the intervention, there was no statistical significance in the overall health score of the quality of life between the observation group and the control group before the intervention (P>0.05). The overall health score of quality of life in the observation group was significantly higher than that in the control group after the intervention, with a statistically significant difference (P<0.05) (Table-II).

**Table-II:** Overall health status score of quality of life before and after the intervention (*χ̅*±*S*).

Group	n	Before intervention	After intervention
Observation group	33	56.97±3.909	65.04±8.569
Control group	33	45.64±3.847	51.30±6.886
t value		0.48	3.13
P value		0.6	0.002

*P<0.05.

According to the results of the skin reaction grading before and after radiotherapy, there were 18 cases (55%) in grade I, 13 cases (39%) in grade II, two cases (0.6%) in grade III and 0 cases in grade IV in the observation group after radiotherapy. In the control group, there were six cases (18%) of Grade-I, 10 cases (30%) of Grade-II, 14 cases (42%) of Grade-III and three cases (9%) of Grade-IV after radiotherapy. The skin reaction of the observation group after radiotherapy was significantly better than that of the control group (Table-III).

**Table-III:** Grading of skin reaction before and after radiotherapy [number of cases (%)].

Group	n	Before radiotherapy	After radiotherapy			

		Grade 0	Grade-I	Grade-II	Grade-III	Grade-IV
Observation group	33	0	18 (55)	13 (39)	2 (0.6)	0
Control group	33	0	6 (18)	10 (30)	14 (42)	3 (9)
c2value		5.15				
P value		<0.01				

*P<0.05.

## DISCUSSION

The results of psychological support intervention on the psychological status of NPC patients during radiotherapy before and after the intervention, there was no statistically significant difference in psychological resilience scores between the observation group and the control group before the intervention (P>0.05), indicating comparability between the two groups. After the intervention, the psychological resilience score of the observation group was significantly higher than that of the control group, with a statistically significant difference (P<0.05). The psychological resilience scores after the intervention were significantly higher in the observation group than before the intervention, and those in the control group were higher than before the intervention, with a statistically significant difference (P<0.05). In this study, the psychological state of NPC patients at each stage was explored by means of psychological intervention, with a view to enhancing patients’ confidence, encouraging them to face treatment actively and reducing the occurrence of skin reactions.

It is an issue that cannot be ignored regarding the psychological health of patients suffering from NPC receiving radiotherapy. Therefore, correct and reasonable psychological attention to patients as well as timely and reasonable psychological intervention are of great necessity. Chen et al.^14^ revealed in his study the positive impact of targeted interventions on patients suffering from NPC, which is consistent with the findings of Xie et al.^15^ Psychological interventions assist NPC patients to face the disease with a positive, optimistic and positive attitude, improve their hope for the future, and encourage them to reduce negative emotions and fight against the disease.

In 2018, there were 129,079 newly diagnosed cases of nasopharyngeal carcinoma(NPC) worldwide, according to data published by World Health Organization(WHO).^16^ Radiotherapy, as the main treatment method for NPC patients, has contributed to a significant increase in the cure rate.^4^,^5^ However, the disease itself and the adverse effects of radiotherapy make skin reactions a common adverse effect after radiotherapy in NPC patients.^17^ It has been reported that most NPC patients choose to give up treatment due to their inability to tolerate the pain of complications during radiotherapy, thus affecting the therapeutic effect and prognosis.

Recent years have witnessed the deepening of studies on NPC, and the quality of life of tumor patients has been reported by more and more literature, becoming one of the hot spots of medical attention.^18^-^20^ Sun et al.^21^ pointed out the positive impact of comprehensive nursing interventions on the improvement of the quality of life of NPC patients, i.e., psychological interventions are of great importance for the consultation of NPC patients. Additional studies^22^,^23^ found that the higher the psychological resilience of NPC patients, the better the self-management efficacy and the disease treatment and recovery, showing a positive correlation.

With the gradual shift from the traditional medical model to the biopsychosocial medical model, healthcare professionals have been further sublimated from the past concept of focusing only on patient treatment outcomes and treatment effects. Hastert et al.^24^ proposed that in addition to assessing the effects of patients suffering from NPC in terms of somatic indicators, attention should also be paid to psychological conditions and social needs, which would also affect the quality of life of tumor patients to varying degrees.

### Limitations of study:

The present study is limited by the small size and the short-term follow-up. In the future, research with a larger sample size and long-term prognosis is required to establish the viability of the combination psychological support intervention and objective study design for the best interest of more patients.

## CONCLUSION

NPC patients with higher mental resilience are more confident in the treatment and recovery of the disease. They are not only willing to actively look for information about the treatment and recovery of the disease, but also can better implement the medical advice and related recovery matters from medical and nursing staff. For this reason, nursing staff should strengthen communication with NPC patients and their families to timely understand the psychological status of patients. In the process of intervention, adequate psychological counseling should be given to NPC patients to relieve their anxiety, depression and other negative emotions, which contributes to reducing the side effects of radiotherapy and helping them establish a positive attitude towards life. In short, psychological interventions will deepen NPC patients’ perception of the disease and thus motivate them to build confidence. In this way, patients are more motivated to participate in the process of treating the disease, thereby improving their quality of life and their recovery.

### Authors’ Contributions:

**CW and XL** carried out the studies, participated in collecting data, and drafted the manuscript, and are responsible and accountable for the accuracy or integrity of the work.

**YL and DL** performed the statistical analysis and participated in its design.

**LY and YW** participated in acquisition, analysis, or interpretation of data and draft the manuscript.

All authors read and approved the final manuscript.
